# Development of the Korean Medicine Core Outcome Set for Facial Palsy: herbal medicine treatment of patients with facial palsy in primary clinics

**DOI:** 10.3389/fmed.2024.1391544

**Published:** 2024-05-22

**Authors:** Soo-Dam Kim, Sungha Kim, Mi Ju Son, Jiyun Cha, Pyung-Wha Kim, Mi Mi Ko, Soobin Jang, Changsop Yang, Myeong Soo Lee

**Affiliations:** ^1^KM Science Research Division, Korea Institute of Oriental Medicine, Daejeon, Republic of Korea; ^2^Digital Health Research Division, Korea Institute of Oriental Medicine, Daejeon, Republic of Korea; ^3^R&D Strategy Division, Korea Institute of Oriental Medicine, Daejeon, Republic of Korea; ^4^Department of Preventive Medicine, College of Korean Medicine, Daegu Haany University, Gyeongsan, Gyeongsangbukdo, Republic of Korea

**Keywords:** core outcome set, facial palsy, primary care, herbal medicine, Korean medicine

## Abstract

**Introduction:**

Facial palsy (FP) significantly affects the quality of life of patients and poses a treatment challenge in primary healthcare settings. This study aimed to develop a Korean medicine (KM) core outcome set (COS) for FP, with a focus on evaluating the effectiveness of herbal medicine (HM) treatments in KM primary clinics.

**Methods:**

Outcomes and effect modifiers related to FP treatments were initially identified through related review articles. Subsequently, experts in the field took part in three rounds of modified Delphi consensus exercises to refine and prioritize these outcomes and effect modifiers. Additionally, primary KM clinicians were involved in a Delphi consensus round to assess the suitability and feasibility of the proposed COS in real-world clinical settings.

**Results:**

The initial review of related literature identified 44 relevant studies, resulting in an initial selection of 23 outcomes and 10 effect modifiers. The expert consensus process refined these to 8 key outcomes and 6 effect modifiers, which established the foundation of the COS-FP-KM. Subsequently, primary KM clinicians confirmed the practicality and applicability of the COS, endorsing its suitability for use in KM primary clinics.

**Conclusion:**

The COS-FP-KM establishes a standardized approach for assessing HM treatment effectiveness in FP patients in KM primary clinics. The COS-FP-KM encourages consistent outcome reporting and enhances patient care quality. Future work should aim to integrate broader stakeholder perspectives to refine and validate the COS further.

## 1 Introduction

Facial Palsy (FP) is a medical condition characterized by the sudden onset of unilateral facial muscle weakness or paralysis, often resulting in significant functional and esthetic impairment (1, 2). While there are various causes for FP, including infections, local inflammation, and malignancy, the most common form is idiopathic facial paralysis, known as Bell’s palsy (3). This condition, which can occur at any age, significantly impacts the physical appearance, emotional well-being, and overall quality of life of patients (4, 5). It often leads to difficulties in facial expression, eating, speaking, and, in severe cases, specific dysfunctions such as the inability to blink or close the eye, and difficulty puckering the lips or raising the mouth corner, thereby profoundly affecting daily activities and social interactions (3, 6). The prevalence of FP varies globally, but it is considered a relatively common neurological disorder, affecting approximately 10–40 per 100,000 individuals annually (3, 6, 7). In conventional medicine, the primary treatments for FP typically involve the use of medications such as corticosteroids and antiviral agents, often complemented by surgical interventions like facial nerve decompression when necessary (7, 8). However, these treatments often have limited efficacy, particularly in cases of severe palsy or delayed treatment initiation, and are associated with potential side effects (8–11). These limitations highlight the need for more comprehensive treatment strategies in managing FP (7).

In several Asian countries, where herbal medicine (HM) plays an integral role in national healthcare, HM has emerged as a promising alternative treatment for FP, specifically targeting idiopathic peripheral FP, also known as Bell’s palsy. Research indicates its efficacy in reducing inflammation, improving symptoms, and providing neuroprotective effects, particularly through the use of compounds like *Radix saposhnikoviae* and *Bombyx batryticatus* (12, 13). Additionally, a study has shown that patients treated with a combination of acupuncture and HM for Bell’s palsy experienced not only faster overall recovery but also shorter periods of initial recovery (14). However, the integration of HM into clinical practice faces challenges due to methodological inconsistencies, primarily the lack of standardized outcome measures (15). This lack of standardization in research methodologies and assessment scales leads to fragmented understanding, data inconsistency, and potential reporting bias. Such disparities complicate the decision-making process for healthcare practitioners and create uncertainties for patients considering alternative or complementary treatments (16).

While there is suggestive evidence of the benefits of HM in treating FP, there is an obvious need for clinical trials rigorously evaluating its efficacy. In South Korea, most Korean Medicine (KM) facilities are primary clinics (17), where conducting extensive clinical trials is challenging due to limited personnel and resources. Consequently, reported studies may often not meet the highest research standards or focus on outcomes that might not be the most relevant. The Korean Medicine Clinical Practice Guideline for Facial Palsy recommends combining HM with steroid therapy (18), but evidence for the efficacy and safety of HM is still insufficient (19). To address this, the Korean government initiated a pilot project in 2020 to support HM treatment for idiopathic peripheral FP in primary clinics, with health insurance covering the costs for 10 days of HM treatment that meets specific diagnostic and quality standards (20). However, a critical limitation of this project is the lack of a standardized outcome model to evaluate the impact of HM on Bell’s palsy.

A Core Outcome Set (COS) comprises a set of vital outcomes, established through consensus among healthcare professionals and patients, which are essential to be assessed and reported in all clinical trials and practices for a specific medical condition (21). It ensures consistency in data collection and enhances the comparability of research findings across different studies (16). Recognizing the necessity for such standardization in the evaluation of HM treatments for Bell’s palsy, this study aims to develop a Core Outcome Set for Facial Palsy in Korean Medicine (COS-FP-KM). The COS-FP-KM will enable the assessment of the effectiveness of HM in treating Bell’s palsy in patients visiting KM primary clinics, using consistent and appropriate evaluation variables. This effort is crucial for improving the quality and relevance of research in this field, thereby facilitating more informed decision-making in clinical practice.

## 2 Methods

In this study, we developed a COS following a structured approach tailored to our specific objectives and scope, as outlined in Table 1. An important aspect of this process was the extraction of effect modifiers, key factors that influence outcomes, to aid clinicians in their history-taking and assessment. Our methodology for developing the COS was aligned with the processes established by the Core Outcome Measures in Effectiveness Trials (COMET) initiative and adhered to standard COS guidelines (22). This development involved three distinct phases, consistent with the methods used in previous studies (23, 24). Initially, we formed a Project Management Group (PMG) responsible for overseeing the development process. This group undertook the task of generating and refining an initial list of outcomes through an extensive literature review. The second phase involved a modified Delphi consensus process conducted by experts in the field. Following this, primary clinicians participated in a similar modified Delphi process during the third phase.

**Table 1:** Recommendations of the core outcome set standards for development (COS-STAD).

Domains	No.	Methodology	Notes
Scope specification	1	The research or practice setting(s) in which the COS is to be applied.	The COS will be applied in research studies for primary KM clinics.
	2	The health condition(s) covered by the COS.	The disease covered by the COS is facial palsy.
	3	The population(s) covered by the COS.	The target population for the COS consists of patients with facial palsy.
	4	The intervention(s) covered by the COS.	The intervention covered by the COS is HM of KM.
Stakeholders involved	5	Those who will use the COS in research.	KM researchers in primary clinics will use the COS for clinical trials.
	6	Healthcare professionals with experience of patients with the condition.	KM specialists and KM clinicians in primary clinics who participated in COS development.
	7	Patients with the condition or their representatives.	Patients did not participate.
Consensus process	8	The initial list of outcomes considered both healthcare professionals’ and patients’ views.	The initial list of outcomes included in the COS is identified through a literature review.
	9	A scoring process and consensus definition were described *a priori*.	A Delphi survey and consensus meeting was adopted to select the outcomes.
	10	Criteria for including/excluding/adding outcomes were described *a priori*.	Delphi with experts: review of all panels, inclusion criteria was unanimity. Delphi with primary clinicians: 9-point Likert scale and calculation of CVR.
	11	Care was taken to avoid ambiguity of language used in the list of outcomes.	The language and medical terms in our COS ensure uniformity of the outcome terms.

COS, core outcome set; CVR, content validity ratio; HM, herbal medicine; KM, Korean medicine.

### 2.1 Phase 1: establishment of project management group and development of an initial list of outcomes and effect modifiers

A PMG was formed for this study, comprising five expert researchers from the Korea Institute of Oriental Medicine. Their responsibilities included an extensive literature review and extracting crucial outcomes and effect modifiers while ensuring the removal of any redundant results. The scope of the COS was determined, followed by a focused review of literature related to FP (Supplementary material 1).

In our analysis, we concentrated on review papers related to HM trials for patients with FP, with a focus on verifying the inclusion criteria and outcomes of the studies incorporated in these reviews. The review papers included a diverse range of studies, including Randomized Controlled Trials (RCTs), observational studies, and case reports. For each study, we extracted key information, including study design, target disease, sample size, and various outcomes. Outcomes were categorized into doctor-reported and patient-reported outcomes, and outcomes associated with medical devices. The process involved meticulous examination and verification of each study’s design and outcomes. Any discrepancies or disagreements encountered during this analytical stage were resolved through discussion among the research team.

Additionally, we reviewed previously published books and articles pertinent to FP, such as “Korean Medicine Clinical Practice Guideline for Facial Palsy” and other relevant literature on risk factors for FP (18, 25). This review helped us extract recommended outcomes and effect modifiers specific to FP. To streamline our research, duplicate studies and outcomes were removed. The PMG meticulously reviewed and refined the list of outcomes and effect modifiers in alignment with the rationale of this COS, finalizing the list for the next phase of the study.

### 2.2 Phase 2: expert consensus process through modified Delphi method

In this phase, we collaborated with The Korean Acupuncture & Moxibustion Medicine Society and the Korean Medicine Clinical Practice Guideline for Facial Palsy Development Team for a modified Delphi round. Following a request from the PMG, these organizations recommended a panel of experts, selected for their extensive experience and expertise in the field of facial palsy treatment. Ultimately, five experts agreed to participate after understanding the objectives and scope of the COS.

The modified Delphi round involved distributing materials for review, followed by group feedback sessions and consensus meetings. In the initial round, panel members were presented with the list of outcomes and effect modifiers identified in Phase 1. The process followed a unanimous decision-making approach, where each item was deliberated upon with the option of “consensus in” or “consensus out” to determine its relevance, and conflicts were resolved through discussion, continuing until unanimous agreement was reached. Additionally, panel members had the opportunity to suggest any new outcomes or effect modifiers not previously listed.

In the two following rounds (Rounds 2 and 3), the panel engaged in detailed discussions on all the proposed outcomes and effect modifiers, including those added in the first round. These discussions considered the initial responses from the panel members and were directed toward ensuring alignment with the scope of the COS. The process was iterative, persisting until a collective agreement was achieved on the final compilation of outcomes and effect modifiers to be included in the COS.

### 2.3 Phase 3: primary clinicians consensus process through modified Delphi method

In this critical phase, primary clinicians, pivotal in the field of FP treatment, were engaged to review the feasibility of previously identified outcomes and effect modifiers. This panel, comprising KM primary clinicians with a minimum of five years of clinical experience, was assembled following recommendations from The Korean Medicine Specialists Association at the request of the PMG. The panel included 11 professionals, with two specializing in facial palsy.

Their task was to assess the practicality of implementing the outcomes and modifiers in KM primary clinic settings. The questionnaire was structured into two sections: the first addressing effect modifiers related to facial palsy, and the second focusing on several distinct outcome measures. These included the House–Brackmann scale, the patient-perceived onset of symptom improvement, the EuroQoL 5-Dimension 5-Level (EQ-5D-5L) & EuroQol Visual Analog Scale (EQ-VAS) assessments for quality of life, and evaluations of patient satisfaction. Each of these outcome measures was rated by the clinicians on a detailed nine-point scale.

Notably, some items such as certain laboratory tests and records of adverse events were not included in this phase. These were deemed essential by the PMG for investigating the safety and effectiveness of herbal medicines in treating facial palsy and were thus considered inherent elements of the COS.

### 2.4 Data analysis

#### 2.4.1 Expert consensus in phase 2

During the expert-led modified Delphi consensus in Phase 2, we set unanimity as the predefined criterion for achieving consensus on the COS. Decisions were classified as either achieving full consensus “consensus in” or not “consensus out.”

#### 2.4.2 Primary clinician consensus in phase 3

In line with our previously published COS studies (23, 24), we employed the Content Validity Ratio (CVR) alongside measures of consensus and convergence to evaluate content validity and the formation of consensus. For the panel of 11 Delphi participants, the critical values for CVR, consensus degree, and convergence were established at ≥0.636, ≥0.75, and ≤ 0.5, respectively (26, 27).

### 2.5 Ethics and consent

The study received an exemption from ethical approval by the Institutional Review Board (IRB) of the Korea Institute of Oriental Medicine in Daejeon, Republic of Korea (IRB approval No. I-2203/003-001). Prior to participation, all panel members were required to provide written informed consent, ensuring their voluntary involvement and understanding of the study’s objectives and procedures.

## 3 Results

### 3.1 Phase 1: development of an initial list of outcomes and effect modifiers

**Figure 1:**
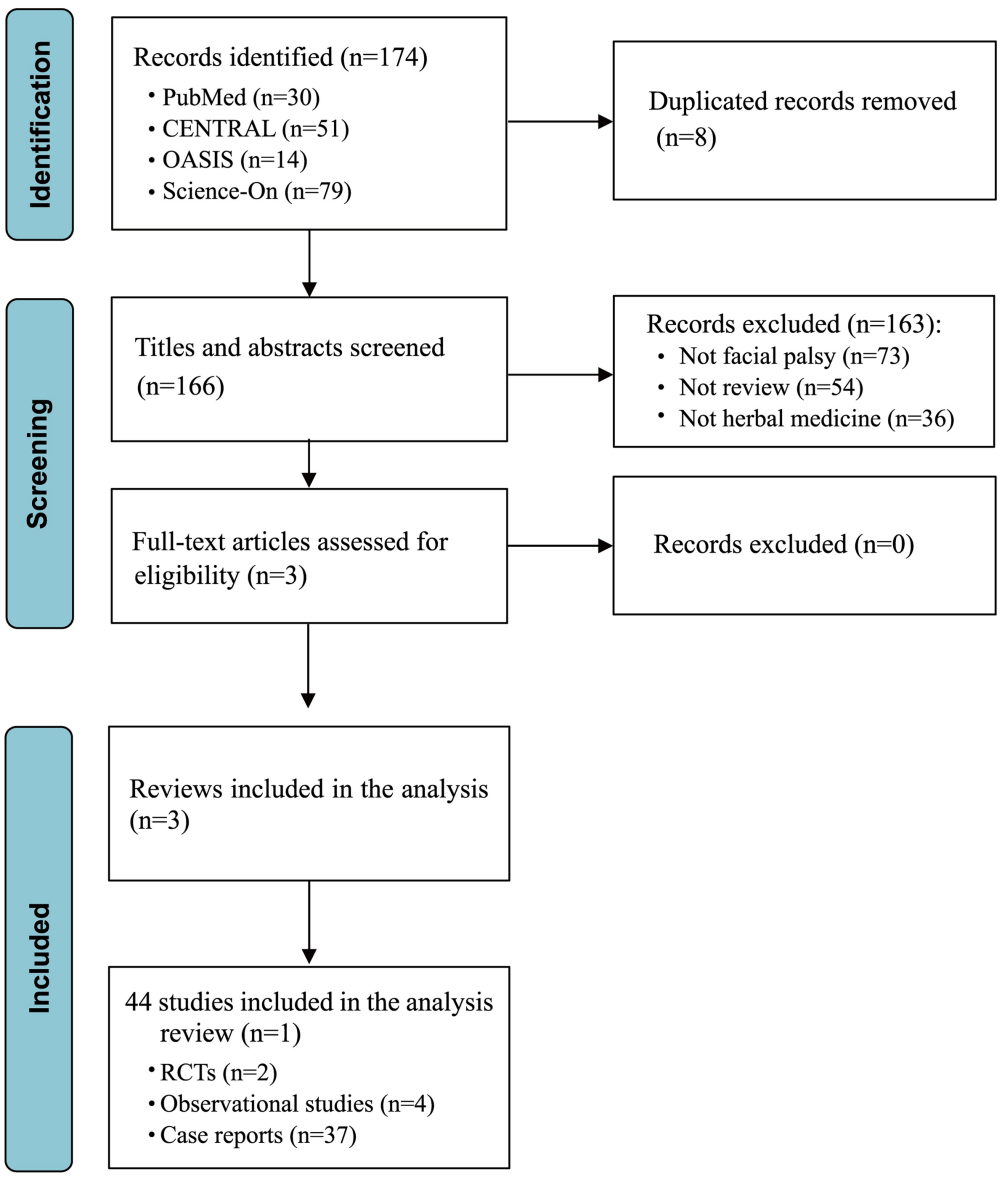
Flow chart of selection process. RCT, randomized controlled trial.

The initial literature search across databases including PubMed, CENTRAL, OASIS, and Science-On yielded a total of 174 studies. Of these, eight were removed due to duplication. Through the screening of titles and abstracts, an additional 163 studies were excluded for not meeting our inclusion criteria. Following a thorough full-text review for eligibility, no additional studies were excluded, resulting in the inclusion of three review studies for detailed analysis (Figure 1). Among these, one review detailed 14 distinct tools for assessing facial palsy, another encompassed two relevant RCTs and four prospective observational studies, and the third review brought in 37 case reports. Consequently, our analysis incorporated a total of 44 studies that were extracted from selected review articles, as enumerated in Supplementary Table S1.

From the three selected review articles, we extracted 44 individual studies, yielding a diverse array of outcomes and effect modifiers relevant to FP. In this step (Step 1), we identified 22 specific outcomes, including several specific scales and assessments for FP, such as the House–Brackmann scale and Yanagihara’s unweighted grading system. Subsequently, an in-depth examination of related literature, including the “Korean Medicine Clinical Practice Guideline for Facial Palsy” and other studies addressing FP risk factors (Step 2) (18, 25), contributed an additional nine outcomes and 10 effect modifiers. Following these stages, the PMG engaged in comprehensive discussions to evaluate these items, categorizing them as suitable (“in”) or not (“out”) for inclusion in the COS for the next phase of the study. After eliminating duplicates from the initial findings, the refined list for the Phase 2 consensus included a total of 23 outcomes and 10 effect modifiers (Table 2).

**Table 2:** Summary of outcomes and effect modifiers identified in Phase 1.

	Item	Selected reviews (Step 1)	Related literature (Step 2)	Deliberations by PMG (Outcomes for Phase 2)
Outcomes	House-Brackmann scale	○		In
	Yanagihara’s unweighted grading system	○		In
	Sunnybrook scale	○		In
	Facial nerve grading system 2.0	○		In
	Nottingham system	○		In
	Stennert’s specific grading system for secondary defect	○		In
	The scale of Peitersen	○		In
	The scale of Murata *et al*	○		In
	Synkinesis assessment questionnaire	○		In
	The scale of Kim for synkinesis	○		In
	Detailed evaluation of facial symmetry	○		Out (Not standardized evaluation)
	Lucille Daniels method	○		Out (Not standardized evaluation)
	Visual analog scale	○		Out (Non-Specific to FP)
	numerical rating scale	○		Out (Non-Specific to FP)
	The evaluation of Na-kamura for synkinesis	○		In
	The evaluation of Haruo Saito for synkinesis	○		In
	The scale of Kim for contracture	○		In
	The scale of Edson Ibrahim Mitrefor facial asymmetry	○		In
	The scale of Scott		○	In
	Proposed comprehensive scale of Bettina Wabbels et al. for the estimation of treatment results of hemifacial spasm		○	In
	Scale for crocodile tear syndrome		○	In
	Scale for quality of life (EQ-5D-5L & EQ-VAS)		○	In
	Satisfaction with treatment		○	In
	Myoneural excitability test	○		Out (Hard to evaluate in KM primary clinics)
	Digital infrared thermography imaging	○		Out (Hard to evaluate in KM primary clinics)
	Electromyography	○		Out (More suitable as effect modifier)
	Electroneurography	○		Out (More suitable as effect modifier)
	Aspartate transaminase		○	In
	Alanine transaminase		○	In
	Blood urea nitrogen		○	In
	Creatinine		○	In
Effect modifiers	Age & Gender		○	In
	Complete paralysis (YES/NO)		○	In
	No recovery within 3 weeks (YES/NO/NA)		○	In
	Postauricular pain (YES/NO)		○	In
	Taste disorder (YES/NO)		○	In
	Ramsay Hunt syndrome (YES/NO)		○	In
	Pregnancy (YES/NO)		○	In
	Diabetes (YES/NO)		○	In
	Severe nerve degeneration on EMG/ENoG (YES/NO/UK)		○	In
	Taking Western medicine for facial palsy (YES/NO)		○	In

FP, facial palsy; KM, Korean medicine; EMG, electromyography; ENoG, electroneurography; EQ-5D-5L, EuroQoL 5-Dimension 5-Level; EQ-VAS, EuroQol visual analog scale.

### 3.2 Phase 2: expert consensus process through modified Delphi method

**Figure 2:**
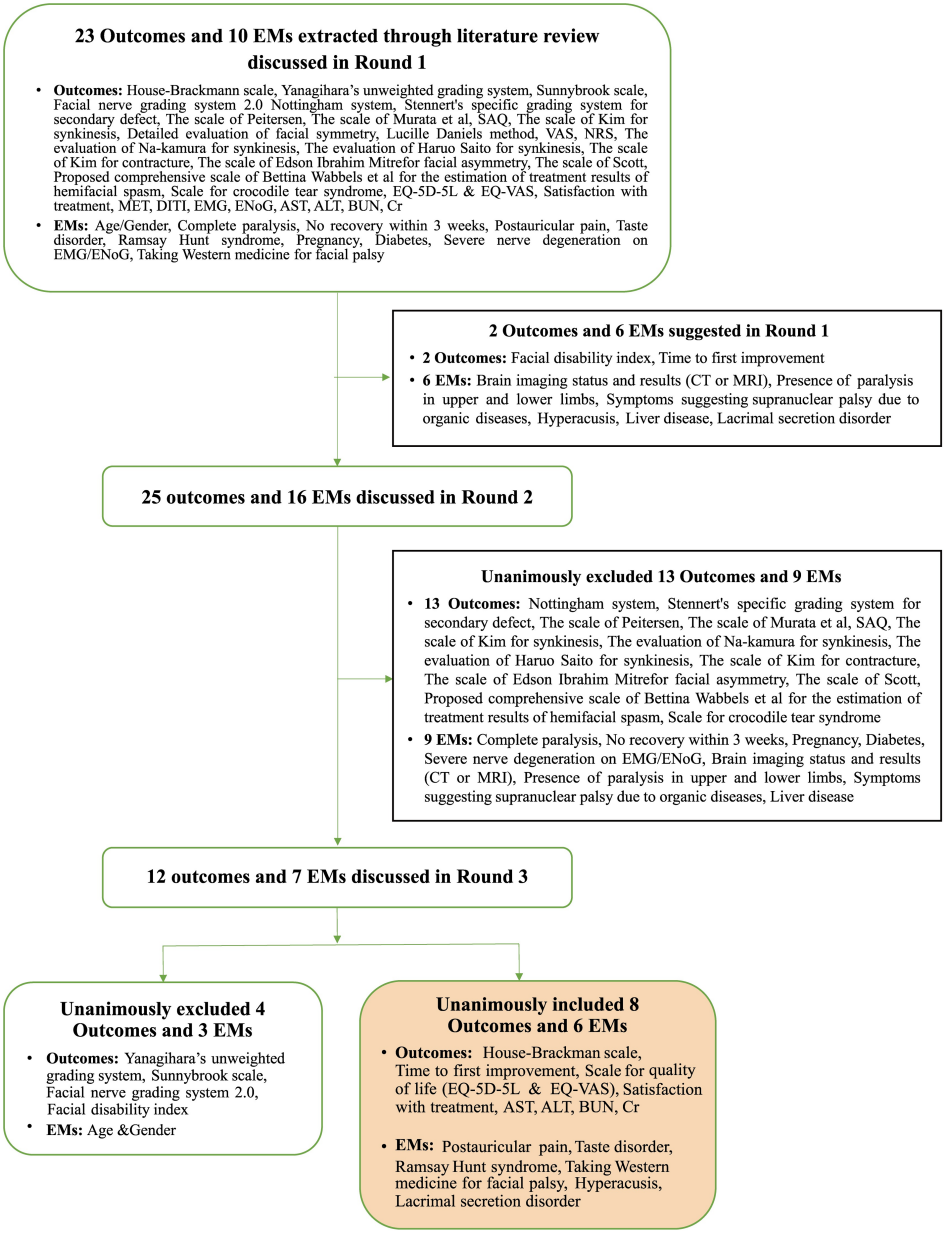
Overview of the consensus process during the three Delphi rounds in Phase 2. EMs, effect modifiers; SAQ, synkinesis assessment questionnaire; VAS, visual analog scale; NRS, numerical rating scale; MET, myoneural excitability test; DITI, digital infrared thermographic imaging; EMG, electromyography; ENoG, electroneurography; AST, aspartate transaminase; ALT, alanine transaminase; BUN, blood urea nitrogen; Cr, creatinine.

In Phase 2, we assembled a panel of five experts, each with a minimum of 10 years of clinical experience in FP and KM, working at facial nerve centers (Supplementary Table S2). These experts actively participated in the initial round of the Delphi process, during which they proposed two new outcomes and six potential effect modifiers. The PMG then evaluated each suggested outcome for potential inclusion in the next round. The questionnaire for the second round included both the newly proposed outcomes and effect modifiers, as well as those carried over from the first round. All participants from Round 1 were invited back. During this round, there was a unanimous agreement to exclude 13 outcomes and nine effect modifiers. Moreover, there was agreement that the items with no consensus would be revisited in Round 3. Accordingly, round 3 questionnaires comprised 12 outcomes and seven effect modifiers without a reached consensus. As in Round 2, all respondents were invited to participate in Round 3. Finally, eight outcomes and six effect modifiers met the consensus inclusion criteria for the COS. Table 3 and Figure 2 present the process involved for each round.

**Table 3:** Summary of the consensus process during the three Delphi rounds in Phase 2.

Category	Item	Result	Note
Outcomes	House-Brackmann scale	In	
	Yanagihara’s unweighted grading system	Out	less aligned with criteria compared to the House-Brackmann scale
	Sunnybrook scale	Out	less aligned with criteria compared to the House-Brackmann scale
	Facial nerve grading system 2.0	Out	less aligned with criteria compared to the House-Brackmann scale
	Facial disability index	Out	Tailored for specific conditions or aspects
	Time to first improvement	In	
	Nottingham system	Out	less aligned with general FP assessment needs
	Stennert’s specific grading system for secondary defect	Out	Tailored for specific conditions or aspects
	The scale of Peitersen	Out	less aligned with general FP assessment needs
	The scale of Murata *et al*	Out	less aligned with general FP assessment needs
	Synkinesis assessment questionnaire	Out	Less correlated with evaluation of FP
	The scale of Kim for synkinesis	Out	Less correlated with evaluation of FP
	The evaluation of Na-kamura for synkinesis	Out	Less correlated with evaluation of FP
	The evaluation of Haruo Saito for synkinesis	Out	Less correlated with evaluation of FP
	The scale of Kim for contracture	Out	Tailored for specific conditions or aspects
	The scale of Edson Ibrahim Mitrefor facial asymmetry	Out	Tailored for specific conditions or aspects
	The scale of Scott	Out	less aligned with general FP assessment needs
	Proposed comprehensive scale of Bettina Wabbels et al. for the estimation of treatment results of hemifacial spasm	Out	less aligned with general FP assessment needs
	Scale for crocodile tear syndrome	Out	Tailored for specific conditions or aspects
	Scale for quality of life (EQ-5D-5L & EQ-VAS)	In	
	Satisfaction with treatment	In	
	Aspartate transaminase	In	
	Alanine transaminase	In	
	Blood urea nitrogen	In	
	Creatinine	In	
Effect modifiers	Age & Gender	Out	Less correlated with effect of HM
	Complete paralysis (YES/NO)	Out	Can be substituted with the House-Brackmann scale
	No recovery within 3 weeks (YES/NO/NA)	Out	Can be substituted with the time to first improvement
	Postauricular pain (YES/NO)	In	
	Taste disorder (YES/NO)	In	
	Ramsay Hunt syndrome (YES/NO)	In	
	Pregnancy (YES/NO)	Out	Not directly influence FP outcomes
	Diabetes (YES/NO)	Out	Not directly influence FP outcomes
	Severe nerve degeneration on EMG/ENoG (YES/NO/UK)	Out	Difficult to assess in KM primary clinic
	Taking Western medicine for facial palsy (YES/NO)	In	
	Brain imaging status and results (CT or MRI)	Out	Difficult to assess in KM primary clinic
	Presence of paralysis in upper and lower limbs	Out	Difficult to assess in KM primary clinic
	Symptoms suggesting supranuclear palsy due to organic diseases	Out	Difficult to assess in KM primary clinic
	Hyperacusis (Yes/No)	In	
	Liver disease (Yes/No)	Out	Not directly influence FP outcomes
	Lacrimal secretion disorder (Increase/Decrease/No)	In	

FP, facial palsy; KM, Korean medicine; EMG, electromyography; ENoG, electroneurography; EQ-5D-5L, EuroQoL 5-Dimension 5-Level; EQ-VAS, EuroQol visual analog scale; CT, computed tomography; MRI, magnetic resonance imaging.

### 3.3 Phase 3: primary clinicians consensus process through modified Delphi method

In this phase, the panel included 11 clinicians, with two specializing in FP (Supplementary Table S3). Primary clinicians reached a consensus on all proposed items, as evidenced by the CVR surpassing the critical threshold for each question. This consensus affirmed that every item was both pertinent for the COS and practicable for data collection within KM primary clinic settings. The evaluation of effect modifiers and patient satisfaction yielded a high degree of consensus and convergence, reflecting a consistent perspective on the inclusion of these effect modifiers. However, agreement on the practicality of including the EQ-5D-5L and EQ-VAS was less definitive, as indicated by a low level of consensus and convergence, highlighting varying views on their integration (Table 4).

**Table 4:** Results of the consensus process in primary clinicians.

Category	Question	Mean	Median	CVR	Degree of consensus	Degree of convergence
Outcomes	Time to first improvement	7.91	8.00	1.00	0.75	1.00
	House-Brackmann scale	7.36	8.00	0.82	0.75	1.00
	Quality of life (EQ-5D-5L&EQ-VAS)	5.36	6.00	0.64	0.58	1.25
	Satisfaction of treatment	7.73	8.00	1.00	0.88	0.50
Effect modifiers	Effect modifiers about related HM treatment for FP; Postauricular pain Taste disorder Ramsay Hunt syndrome Taking Western medicine for facial palsy Hyperacusis Lacrimal secretion disorder	7.27	7.00	1.00	0.86	0.50
	Postauricular pain related items Presence of postauricular pain (Yes/No) Duration of postauricular pain (Day) Severity of postauricular pain (VAS)	7.45	8.00	0.82	0.75	1.00

CVR, critical value of ≥ 0.636—suitable for 11 panelists—was judged to suggest consensus among the panelists. A degree of consensus of ≥ 0.75 and a degree of convergence of ≤ 0.5 were judged to indicate that agreement among panel experts was achieved. EQ-5D-5 L, EuroQoL 5-Dimension 5-Level; EQ-VAS, EuroQol visual analog scale; HM, herbal medicine; FP, facial palsy; VAS, visual analog scale.

## 4 Discussion

In the process of creating a COS for HM treatments targeting patients with FP in primary KM clinics, we adhered to the guidelines provided by the COMET initiative (22). This endeavor began with a review of related literature undertaken by the PMG (Phase 1). Subsequent phases involved modified Delphi exercises with experts in FP and KM (Phase 2), and a final phase with clinicians from KM primary clinical settings (Phase 3). These exercises were pivotal in assessing the applicability and feasibility of the COS, culminating in the identification of eight specific outcomes and six effect modifiers for the final COS.

In the development process from Phase 1 to Phase 3, our research was oriented toward understanding the distinct characteristics of FP and its practicality within primary KM clinical settings. FP often manifests suddenly and significantly impacts patient quality of life by affecting facial expressions and functions (1–5). While treatments including corticosteroids, antiviral agents, and HM are typically effective within three weeks to three months (28, 29), around 30% of patients may still suffer from enduring complications such as permanent facial weakness, even after undergoing proper treatment (30). During the consensus process, the PMG and experts agreed on the importance of considering a broad range of physical and psychological factors in the COS for FP. This perspective was crucial, given that FP symptoms can vary widely in severity and impact, and are influenced by an array of external and internal factors.

The applicability of the COS in primary KM clinics was also a key consideration, recognizing that these clinics often have limited resources and personnel for conducting large trials, as previously noted in our studies (23, 24). Therefore, we emphasized the feasibility of implementing the COS in primary KM clinical settings. Drawing from previous studies in KM clinics (7, 29), our goal was to develop a practical and effective COS with simple, accurate outcomes that seamlessly integrate into routine clinical practices without significant disruption. In developing the COS-FP-KM, we paid special attention to selecting outcomes that align with the practical realities of these clinical settings. Considering that a significant portion of KM institutions operates as primary care clinics (17), it was essential to choose outcomes that are feasible to assess within these resource-limited environments.

The COS outcomes for FP, including the House–Brackmann scale, time to first improvement, quality of life scales (EQ-5D-5L and EQ-VAS), and patient satisfaction measures, were selected for their straightforward and efficient applicability. These outcomes were designed to facilitate treatment effectiveness assessments without overburdening clinicians with additional workload or resource demands. Crucially, this approach of reducing clinical workload enhances decision-making quality, as it allows clinicians to concentrate more on essential patient care aspects, thereby streamlining the evaluation process and ultimately leading to better patient outcomes (31, 32). This strategy not only simplifies assessments but also boosts the overall efficacy and efficiency of treatments in KM clinics. Additionally, the inclusion of biochemical parameters such as Aspartate Aminotransferase, Alanine Aminotransferase, Blood Urea Nitrogen, and Creatinine, as well as the monitoring of Western medicine usage, are crucial for evaluating the safety of HM treatments. These outcomes are essential for simultaneously assessing the efficacy and potential side effects of HM, enabling the monitoring of adverse reactions or complications that may arise from HM treatments or their interactions with conventional medications (33, 34). Furthermore, including effect modifiers like postauricular pain, taste disorder, and Ramsay Hunt syndrome in the COS for FP is considered for its potential impact on treatment outcomes and patient prognosis (35). These frequently observed symptoms in FP provide insight into the unique experiences of each patient. This approach leads to the development of tailored and more effective treatment strategies within KM Clinics, acknowledging the diverse manifestations of FP.

While our study is extensive, it faces several limitations impacting the derived outcomes. In the initial literature review (Phase 1), we only included three review papers, potentially overlooking some other relevant trials. This approach might have led to an incomplete coverage of relevant trials, thereby potentially limiting the range of outcomes in our COS for FP. Furthermore, the process of developing the COS encountered a limitation in the panel diversity. Thus, the number of panelists, sourced from a professional society, was relatively smaller than that in comparable studies (36, 37), which could have restricted the variety of insights during the COS development. Additionally, our study primarily involved disease specialists and primary KM clinicians, omitting participation from other vital stakeholders like patients and policy experts. While we attempted to mitigate this shortcoming by having clinicians apply the COS in practical settings and gather patient feedback, a more comprehensive approach involving semi-structured interviews with a broader stakeholder group is recommended for future refinements of the COS.

## 5 Conclusion

COS-FP-KM is the first COS on FP developed for primary KM care settings. The new tool is expected to standardize outcome selection and reporting, aiding in an efficient evaluation of the therapeutic effects of HM for FP. This COS will be used in the the Korean government HM pilot project, with continuous discussions to enhance the degree of completeness and reliability. Further studies are warranted to involve more relevant stakeholder groups, such as patient representatives and policy experts.

## Data availability statement

The original contributions presented in the study are included in the article/Supplementary material, further inquiries can be directed to the corresponding author.

## Ethics statement

Ethical review and approval was not required for the study on human participants in accordance with the local legislation and institutional requirements. Prior to participation, all panel members were required to provide written informed consent, ensuring their voluntary involvement and understanding of the study's objectives and procedures.

## Author contributions

S-DK: Writing – original draft, Writing – review & editing, Formal analysis, Visualization. SK: Writing – original draft, Writing – review & editing, Conceptualization, Data curation, Funding acquisition, Investigation, Methodology, Project administration, Resources, Supervision, Validation. MJS: Methodology, Resources, Writing – original draft, Writing – review & editing. JC: Methodology, Writing – original draft, Writing – review & editing. P-WK: Methodology, Writing – original draft, Writing – review & editing. MMK: Writing – original draft, Writing – review & editing, Formal analysis. SJ: Writing – original draft, Writing – review & editing, Methodology. CY: Methodology, Writing – original draft, Writing – review & editing, Funding acquisition, Project administration. ML: Methodology, Writing – original draft, Writing – review & editing, Conceptualization, Supervision.
