# Supplementary material for: Development of the Korean Medicine Core Outcome Set for Facial Palsy: herbal medicine treatment of patients with facial palsy in primary clinics
Source: Front Med (Lausanne). 2024 May 22;11:1391544. doi: 10.3389/fmed.2024.1391544 (PMC11150695; doi:10.3389/fmed.2024.1391544)
Supplement: Supplementary file 1 [file Table_1.DOCX]

**Supplementary 1**. Summary of literature review

**1. Methods**

**1.1. Searched databases:** MEDLINE (via PubMed), and the Cochrane Central Register of Controlled Trials (CENTRAL), Oriental Medicine Advanced Searching Integrated System [OASIS], and Science-On

**1.2. Searching date:** from inception to Sep 15, 2021

**1.3. Search strategies**

*1.3.1. Medline (via PubMed)*

- ("facial nerve diseases"[MeSH Terms] OR ("facial"[All Fields] AND "nerve"[All Fields] AND "diseases"[All Fields]) OR "facial nerve diseases"[All Fields] OR ("bell palsy"[MeSH Terms] OR ("bell"[All Fields] AND "palsy"[All Fields]) OR "bell palsy"[All Fields]) OR ("facial paralysis"[MeSH Terms] OR ("facial"[All Fields] AND "paralysis"[All Fields]) OR "facial paralysis"[All Fields]) OR "hemifacial"[All Fields] OR ("spasm"[MeSH Terms] OR "spasm"[All Fields] OR "spasms"[All Fields]) OR ("bell*"[All Fields] OR "facial*"[All Fields] OR "hemifacial*"[All Fields] OR "cranial*"[All Fields] OR "pals*"[All Fields] OR "paralys*"[All Fields] OR "paresi*"[All Fields] OR "spasm*"[All Fields])) AND ("Herbal medicine"[MeSH Terms] OR "plants, medicinal"[MeSH Terms] OR "medicine, traditional"[MeSH Terms] OR "drugs, chinese herbal"[MeSH Terms] OR "medicine, korean traditional"[MeSH Terms] OR "medicine, kampo"[MeSH Terms] OR "medicine, chinese traditional"[MeSH Terms] OR "Plant extracts"[Title/Abstract]) AND "systematic review"[Filter]

*1.3.2. Cochrane Central Register of Controlled Trials (CENTRAL)*

#1 facial nerve diseases

#2 bell palsy

#3 facial paralysis

#4 hemifacial

#5 #1 OR #2 OR #3 OR #4

#6 herbal medicine

#7 Plants, Medicinal

#8 Medicine, Traditional

#9 Drugs, Chinese Herbal

#10 Medicine, Korean Traditional

#11 Medicine, Kampo

#12 Medicine, Chinese Traditional

#13 Plant Extracts

#14 #6 OR #7 OR #8 OR #9 OR #10 OR #11 OR #12 OR #13

#15 systematic review

#16 #5 AND #14 AND #15

*1.3.3. OASIS*

- ‘(한약 OR 첩약 OR 탕 OR 환 OR 산) AND 안면신경마비

*1.3.4. Science-On*

- 전체=한약*|Herb*|traditional|*탕|*환|*산| AND 전체=안면신경마비|안면마비|"facial nerve diseases"|"bell palsy"|"facial paralysis"|hemifacial AND 전체=고찰|"systematic review"

**1.4. Inclusion criteria:**

- Participants: patients with facial palsy

- Intervention: orally administered herbal medicine (except for pharmacoacupuncture)

- Outcomes: effectiveness (symptoms, quality of life, physical examination), safety (laboratory test, physical examination)

- Study design: review, systematic review

| Authors (year) (ref) | Study design | Target disease | Sample size | Outcomes | | |
| --- | --- | --- | --- | --- | --- | --- |
|  |  |  |  | Doctor reported | Patient reported | Medical device |
| Suk (2014) (1) | review | facial palsy, sequelae of facial palsy | n.r. | HBGS, YUGS, FNGS, SFGS, Nottingham system, The scale of Peitersen, Murata scale, The scale of Kim for synkinesis | Stennert scale, SAQ | The evaluation of Haruo Saito for synkinesis, The evaluation of Nakamura for synkinesis, The scale of Kim for contracture, The scale of Edson Ibrahim Mitre for facial asymmetry |
| Li (2015)^†^ (2) | RCT | peripheral facial paralysis | 68/68 |  |  |  |
| Qiao (2014)^†^ (3) | RCT | Bell’s palsy | 28/28 |  |  |  |
| Cho (2008)^†^ (4) | Observational study | acute Bell’s palsy | 65 | HBGS, YUGS |  |  |
| Kim (2001)^†^ (5) | Observational study | Bell’s palsy | 30 | HBGS, DEFS |  |  |
| Kwon (2008)^†^ (6) | Observational study | Bell’s palsy | 30 | HBGS |  |  |
| Park (2004)^†^ (7) | Observational study | Bell’s palsy | 41 | YUGS |  |  |
| Ahn (2007)^‡^ (8) | Case report | facial nerve paralysis caused by traumatic temporal bone fracture | 1 | HBGS, YUGS |  | DITI, EMG |
| Cho (2000)^‡^ (9) | Case report | facial palsy with paralytic strabismus | 1 |  | Lucille Daniels method | MET |
| Cho (2010)^‡^ (10) | Case report | facial nerve palsy | 6 | HBGS, YUGS |  |  |
| Choi (2002)^‡^ (11) | Case report | facial nerve paralysis caused by traumatic temporal bone fracture | 1 | HBGS, YUGS |  | DITI |
| Choi (2003)^‡^ (12) | Case report | Bell’s palsy during pregnancy | 1 |  |  | DITI |
| Choi (2012)^‡^ (13) | Case report | facial nerve palsy in herpes zoster oticus | 1 | YUGS | VAS |  |
| Chu (2009)^‡^ (14) | Case report | facial nerve palsy | 2 | HBGS |  | DITI |
| Ha (2007)^‡^ (15) | Case report | bilateral facial nerve palsy | 1 | HBGS, YUGS |  |  |
| Jang (2013)^‡^ (16) | Case report | bilateral facial nerve palsy | 1 | HBGS |  |  |
| Jang (2016)^‡^ (17) | Case report | facial nerve paralysis caused by traumatic temporal bone fracture | 1 | HBGS, YUGS | NRS | DITI |
| Jeong (2012)^‡^ (18) | Case report | facial paralysis after acoustic neuroma surgery | 1 | YUGS |  |  |
| Jeong (2013)^‡^ (19) | Case report | peripheral facial paralysis | 2 | HBGS, YUGS | NRS |  |
| Jo (2016)^‡^ (20) | Case report | facial nerve palsy in herpes zoster oticus | 1 | HBGS, YUGS, SFGS |  |  |
| Kang (2009)^‡^ (21) | Case report | facial nerve palsy in herpes zoster oticus | 1 | HBGS, YUGS | VAS |  |
| Kang (2010)^‡^ (22) | Case report | facial nerve palsy in herpes zoster oticus in pregnancy | 1 | YUGS | VAS |  |
| Gang (2016)^‡^ (23) | Case report | bilateral facial palsy accompanied by contralateral otitis media | 1 | HBGS, YUGS |  |  |
| Kim (2001)^‡^ (24) | Case report | facial palsy | 5 |  | Lucille Daniels method |  |
| Kim (2002)^‡^ (25) | Case report | bilateral facial palsy | 1 | HBGS |  |  |
| Kim (2003)^‡^ (26) | Case report | Bell’s palsy with trigeminal neuralgia | 1 | YUGS | VAS |  |
| Kim (2003)^‡^ (27) | Case report | nuclear facial nerve paralysis | 1 | YUGS |  |  |
| Kim (2005)^‡^ (28) | Case report | facial paralysis | 1 |  |  |  |
| Kim (2005)^‡^ (29) | Case report | facial nerve palsy | 7 | HBGS |  |  |
| Kim (2006)^‡^ (30) | Case report | facial palsy following microvascular decompression | 3 | HBGS, YUGS |  | ENoG |
| Kim (2016)^‡^ (31) | Case report | facial palsy with a cavernous malformation | 1 | YUGS |  |  |
| Ko (2004)^‡^ (32) | Case report | One-and-a-half syndrome with facial palsy | 1 |  |  |  |
| Lee (2005)^‡^ (33) | Case report | Bell's palsy during pregnancy | 1 | HBGS |  |  |
| Lee (2006)^‡^ (34) | Case report | facial nerve palsy | 12 | HBGS |  |  |
| Lee (2006)^‡^ (35) | Case report | Bell’s palsy caused by trauma | 2 | YUGS |  |  |
| Lee (2009)^‡^ (36) | Case report | Bell s palsy during chemotherapy | 3 | YUGS |  |  |
| Lee (2013)^‡^ (37) | Case report | Peripheral facial palsy in children | 5 | HBGS |  | ENoG |
| Lee (2014)^‡^ (38) | Case report | Soyang type peripheral facial palsy | 2 | HBGS, YUGS | NRS |  |
| Lee (2015)^‡^ (39) | Case report | sequelae of facial palsy | 1 | HBGS |  |  |
| Lee (2017)^‡^ (40) | Case report | Bell's palsy with chronic suppurative otitis media | 1 | HBGS, YUGS | VAS |  |
| Oh (2009)^‡^ (41) | Case report | Bell's palsy | 2 | HBGS, YUGS, | VAS |  |
| Park (2002)^‡^ (42) | Case report | bilateral facial palsy | 1 | HBGS, DEFS |  |  |
| Shin (2008)^‡^ (43) | Case report | Bell’s palsy during pregnancy | 2 | YUGS |  |  |
| Son (2011)^‡^ (44) | Case report | peripheral facial palsy | 2 | HBGS |  |  |
| ^†^Studies were included in Oh (2020) (45), and ^‡^Jeong (2018) (46).  DEFS, detailed evaluation of facial symmetry; DiTi, Digital Infrared Thermography Imaging; EMG, electromyography; ENoG, electroneurography; FNGS, Facial nerve grading system; HBGS, House Brackmann grading system; MET, myoneural excitability test; NRS, numerical rating scale; SAQ, Synkinesis assessment questionnaire; SFGS, Sunnybrook facial grading system; VAS, visual analog scale; YUGS, Yanagihara’s unweighted grading system | | | | | | |

**Table S1.** Characteristics of included studies for facial palsy.

**Reference**

1. Suk KH, Ryu HK, Goo BH, Lee JH, Ryu SH, Lee SY, et al. A Review Study and Proposal of Facial Palsy Sequelae Evaluating Scale. The Acupuncture 2014;31(4):99-108.

2. Li J. Clinical observation on treating peripheral facial paralysis with the Integrative Medicine. Clinical Journal of Chinese Medicine. 2015;7(31):27-9.

3. Qiao RT, Yan Y. Zhong xiyi jiehe zhiliao beier mabi 56 li linchuang fenxi. JOURNAL OF TAISHAN MEDICAL COLLEGE 2014;35(05):432-3.

4. Cho KH, Jung WS, Hong JW, Hwang JW, Na BJ, Park SU, et al. The effectiveness of oriental medical therapy compared to oriental-western medical therapy on acute bell's palsy. J Korean Oriental Med 2008;29(1):146-55.

5. Kim NO, Chae SJ, Son SS. Comparative clinical study between oriental medicine and oriental-western medicine treatment of Bell's palsy. The Journal of Korean Acupuncture & Moxibustion Society. 2001;18(5):99-108.

6. Kwon N-h, Shin Y-j, Kim C-y, Koh P-s, Yi W-i, Joh B-j, et al. Comparative clinical study between oriental medical and oriental-western medical treatment on Bell's palsy. The Journal of Korean Acupuncture & Moxibustion Society. 2008;25(3):19-28.

7. Park IB, Kim SW, Lee CW, Kim HG, Heo SW, Youn HM, et al. Comparative clinical study between oriental medicine and oriental-western medicine treatment on bell's palsy. Journal of Acupuncture Research. 2004;21(5):191-203.

8. Ahn H, Shin M. A Case Report of a Patient with Facial Nerve Paralysis Caused by Traumatic Temporal Bone Fracture. J Oriental Rehab Med. 2007;17(1):159-66.

9. Cho JH, Kim YB, Chae BY. A Casuistics of a Patient with Facial Palsy and Paralytic Strabismus. The Journal of Oriental Medical Surgery, Ophthalmology & Otolaryngology. 2000;13(2):152-64.

10. Cho A, Kim J, Chou C, Won J, Kim C. The changes of Facial nerve palsy by Miso Facial Acupuncture on Orbicularis Oculi muscle : A case study. The Journal of Korean Oriental Medical Ophthalmology & Otolaryngology & Dermatology. 2010;23(2):196-205.

11. Choi SW, Roh JD, Shin MS, Seol H, Song BY, Yook TH. Clinical Study of Patient with Facial Nerve Paralysis Caused by Traumatic Temporal Bone Fracture. The Journal of Korean Acupuncture & Moxibustion Society. 2002;19(3):207-15.

12. Choi S, Park YE, Park YN, Ryoo KS. Clinical study on one case of the Bell`s palsy during the period of pregnancy. THE JOURNAL OF ORIENTAL OBSTETRICS & GYNECOLOGY. 2003;16(3):227-34.

13. Choi EY, Noh KH, Baek JH, Ko MJ. A case report of facial nerve palsy in herpes zoster oticus and postherpetic neuralgia by acupuncture. The Journal of East-West Medicine. 2012;37(4):49-56.

14. Chu M, Jo H, Choi J, Kim S, Park K, Cho G, et al. Clinical Case Study of Facial Nerve Palsy Using Facial Acupuncture. Korean J Oriental Physiology & Pathology. 2009;23(5):1188-92.

15. Ha S, Kim H, Song I, Kim K. A Clinical Case Report of Bilateral Facial Nerve Palsy. J Korean Oriental Pediatrics. 2007;21(3):1-10.

16. Jang S, Jung Y, Lee J, Park S, Kwon H, Shin H. One Clinical Case Report of Bilateral simultaneous facial nerve palsy (Bell's palsy). The Journal of East-West Medicine. 2013;38(1):9-19.

17. Jang Y, Yang T, Shin J, Kim H, Kim T, Jeong M, et al. Clinical Case Study of Facial Nerve Paralysis with Sensorineural Hearing Loss and Tinnitus Caused by Traumatic Temporal Bone Fracture. The acupuncture. 2016;33(1):95-101.

18. Jeong S, Lee H, Park H, Kim Y, Yoo H, Jo H, et al. Clinical Study of 1 Case of Facial Paralysis after Acoustic Neuroma Surgery using Needle-Embedding Therapy. Korean J Oriental Physiology & Pathology 2012;26(6):976-9.

19. Jeong HY, Lee SJ, Ham SH, Lim EC. 2 Case Study Reportings Using Hyeongbangjihwang-tang of a Soyangin Patient Diagnosed with Peripheral Facial Paralysis. J Sasang Constitut Med. 2013;25(2):124-33.

20. Jo S, Lee H. One Case of Facial Nerve Palsy in Herpes Zoster Oticus Treated with electromagnetic therapy stimulator(Whata153). Dae Jeon Dae Hag Gyo Han Ui Hag Yeon Gu So Non Mun Jib. 2016;25(1):53-62.

21. Kang R, Kim H, Han H, Park E, Jang J, Kang H, et al. 1 Case of the facial nerve palsy in Herpes Zoster Oticus - Focused on a case that were improved slightly by medical treatments. THE KOREAN JOURNAL OF ORIENTAL MEDICAL PRESCRIPTION. 2009;17(2):215-24.

22. Kang M. A Clinical Study on the Case of Herpes Zoster Otiucus Occurred in Pregnancy Treated with Bee Venom Pharmarcopuncture. The Journal of Korean Acupuncture & Moxibustion Society. 2010;27(2):155-60.

23. Gang B, Kim H, Lim G, Choi J, Park S, Jung M, et al. A Clinical Experience of Bilateral Facial Palsy accompanied by Contralateral Otitis Media under Treatment for Bell's palsy. J Korean Med Ophthalmol Otolaryngol Dermatol. 2016;29(4):218-31.

24. Kim CH, Kim HG. The clinical case study of facial palsy. The Journal of Oriental Medical Surgery, Ophthalmology & Otolaryngology. 2001;14(2):262-70.

25. Kim MJ, Park SD, Lee AR, Kim KH, Jang JH, Kim KS. Clinical Observation on 1 Case of Patient with Bilateral Facial Palsy. The Journal of Korean Acupuncture & Moxibustion Society. 2002;19(2):238-50.

26. Kim MS, Lee EY. A case report of the Bell's palsy patient which accompanies trigeminal neuralgia. The Journal of Clinical Thesis Korean Acupuncture & Moxibustion Society. 2003(1):46-52.

27. Lee TH, Kim BS, Lim HY, Kim SM, Park JH. A Clinical Report on One Case of Nuclear Facial Nerve Paralysis. Korean J Orient Int Med. 2003;24(2):395-401.

28. Kim GJ, Song HS. A Case Report of Facial Paralysis, the Treatment Acupuncture. Han Bang Chuk Chu Gwan Jeol Hag Hoe Ji. 2005;02(01):55-7.

29. Kim S-J, Sul J-U, Shin M-S, Kim S-J, Choi J-B, Park H-B. The Treatment of Facial Nerve Palsy Using Taping Therapy: Case Report. J Oriental Rehab Med. 2005;15(04):147-55.

30. Kim J, Choi Y, Kim H, Kim J, Lee R-m, Kim K, et al. The Clinical Observation on 3 Cases of Facial Palsy Following Microvascular Decompression in Hemifacial Spasm Patients. The Journal of Korean Acupuncture & Moxibustion Society. 2006;23(6):229-38.

31. Kim S, Noh H, Yi C, Ha Y, Choi D. A Case Report of a Facial Palsy Patient with a Cavernous Malformation. J Int Korean Med. 2016;37(5):815-21.

32. Kim KO, Ko ES, Shin YW. A case study of One-and-a-half syndrome with facial palsy. The Journal of Korea CHUNA Manual Medicine. 2004;5(1):101-7.

33. Lee JA, Ban HR, Cho SH. The case study on one case of the Bell`s palsy during the period of pregnancy. THE JOURNAL OF ORIENTAL OBSTETRICS & GYNECOLOGY. 2005;18(2):186-94.

34. Lee D, Na G, Chiang S, Jeong J, Wei T, Yoon Y. 12 Cases Study of Facial Nerve Palsy Using Oriental Medical Treatment with Taping Therapy. Korean J Oriental Physiology & Pathology. 2006;20(4):1078-84.

35. Lee J, Kim E, Song H, Go S, Kim S, Kim J, et al. Clinical Study of Two Patients with Deveation of the Eye and Mouth Caused by Trauma. The Journal of Korean Acupuncture & Moxibustion Society. 2006;23(4):81-9.

36. Lee S, Lee H, Kang J, Kim Y, KimJungho, Kim N, et al. Effect of Acupuncture Complex Therapy on Three Patients with Bell's Palsy During Chemotherapy. The Journal of Korean Acupuncture & Moxibustion Society. 2009;26(4):107-14.

37. Lee J, Yu S, Lee S. Five Case Reports on Peripheral Facial Palsy in Children. J Pediatr Korean Med. 2013;27(3):20-8.

38. Lee SJ, Cho HW, Jeong HY, Lim EC. 2 Case Study Reportings Using Hyeongbangdojok-san of a Soyangin Patient Diagnosed with Peripheral Facial Palsy. J Sasang Constitut Med. 2014;26(4):389-99.

39. Lee E, Kim S, Kwon M, Shin H, Koh Y, Kang S, et al. Case Study of a Patient with Sequelae of Facial Palsy. J Physiol & Pathol Korean Med. 2015;29(4):347-51.

40. Lee H, Park S. A Case Report of Bell's palsy with Chronic suppurative otitis media treated with Korean Medicine. Herbal formula science. 2017;25(1):115-22.

41. Oh HJ, Song HS. Effect of Bee Venom Pharmacopuncture Therapy on the severe pain back of the ear in patient diagnosed with Bell's palsy. Journal of Korean pharmacopuncture institute. 2009;12(3):81-8.

42. Park YC, Chae SJ, Chae JS, Son SS, Choe IS, Song WS. The clinical study on a case of bilateral facial palsy. The Journal of The Korea Institute of Oriental Medical Informatics. 2002;8(3):103-12.

43. Shin S, Lim H, Lee J, Yoo D. 2 Cases Report of the Bell's Palsy Occurred during Pregnancy. THE JOURNAL OF ORIENTAL OBSTETRICS & GYNECOLOGY. 2008;21(4):258-68.

44. Son SS. 2 sequela of peripheral facial palsy cases treated with Dokwhaljiwhang-tang. Han Bang Chuk Chu Gwan Jeol Hag Hoe Ji. 2011;8(1):1-6.

45. Oh DY, Lee SJ, Park JE, Lee MC, Jeon MK, Park CW, et al. Korean-Western Integrative Medicine for Bell’s Palsy: A Review of Randomized Controlled Trials. J Acupunct Res. 2020;37(4):233-40.

46. Jeong HI, Kim KH, Oh YT, Choi YM, Song BY, Kim JU, et al. Korean Medicine for Treating Facial Palsy - A Literature Review of Case Reports. Journal of Pharmacopuncture 2018;21(4):214-25.
