# Supplementary material for: Development of the Korean Medicine Core Outcome Set for Facial Palsy: herbal medicine treatment of patients with facial palsy in primary clinics
Source: Front Med (Lausanne). 2024 May 22;11:1391544. doi: 10.3389/fmed.2024.1391544 (PMC11150695; doi:10.3389/fmed.2024.1391544)
Supplement: Supplementary file 2 [file Table_2.DOCX]

**Table S2.** Delphi Panel Members of Expert Group

| Expert Group (*n*=5) | *n* (%) |
| --- | --- |
| Specialty | |
| KM internal medicine | 1 (20.0) |
| Acupuncture & Moxibustion medicine | 4 (80.0) |
| Years of FP practice | |
| ≤ 20 | 2 (40.0) |
| 21-30 | 2 (40.0) |
| > 30 | 1 (20.0) |
| Education | |
| PhD | 5 (100.0) |
| Participation in the development of KM clinical practice guidelines for FP | |
| Yes | 3 (60.0) |
| No | 2 (40.0) |

FP, Facial Palsy; KM, Korean Medicine
