# Supplementary material for: Development of the Korean Medicine Core Outcome Set for Facial Palsy: herbal medicine treatment of patients with facial palsy in primary clinics
Source: Front Med (Lausanne). 2024 May 22;11:1391544. doi: 10.3389/fmed.2024.1391544 (PMC11150695; doi:10.3389/fmed.2024.1391544)
Supplement: Supplementary file 3 [file Table_3.DOCX]

**Table S3.** Delphi Panel Members of Primary Clinicians

| Primary Clinicians (*n*=11) | *n* (%) |
| --- | --- |
| Specialty | |
| KM internal medicine | 4 (36.3) |
| Acupuncture & Moxibustion medicine | 2 (18.2) |
| Gynecology | 2 (18.2) |
| General Practitioner | 3 (27.3) |
| Years of FP practice | |
| 5-10 | 7 (63.6) |
| 10-15 | 3 (27.3) |
| 15-20 | 1 (9.1) |
| Workplace | |
| Primary Clinic | 7 (63.6) |
| Public health center | 2 (18.2) |
| KM Hospital | 2 (18.2) |

FP, Facial Palsy; KM, Korean Medicine
